# Editorial: Lighting up cellular dynamics with fluorescent biosensors: design and applications in pathophysiology

**DOI:** 10.3389/fphys.2024.1517919

**Published:** 2024-11-13

**Authors:** Kazunori Kanemaru, Mariangela Panniello, Bianca Calì, Chiara Nardin

**Affiliations:** ^1^ Department of Physiology, Nihon University School of Medicine, Tokyo, Japan; ^2^ Optical Approaches to Brain Function Laboratory, Istituto Italiano di Tecnologia, Genova, Italy; ^3^ Institute of Oncology Research (IOR), Bellinzona and Faculty of Biomedical Sciences, Università della Svizzera Italiana, Lugano, Switzerland

**Keywords:** optical sensing, fluorescence microscopy, optogenetics, signalling pathways, transgenic mouse models, second messengers, spatiotemporal resolution, real-time imaging

## Introduction

Fluorescent biosensors have transformed our understanding of cellular and molecular processes by enabling real-time visualization of biomolecular interactions in living cells. These sensors, combined with a range of microscopy techniques, allow tracking of intracellular and intercellular signalling mechanisms, providing unprecedented insight into the dynamics of physiological and pathological processes. The development of new biosensors and the crucial advances in imaging techniques have paved the way for increasingly detailed spatiotemporal analyses of cellular signalling *in vitro* and *in vivo*.

The collection of articles in this Research Topic highlights recent advances in light-emitting biosensor technology and their application to diverse biological systems. The power of these probes lies in their ability to track a broad range of molecules, including ions, metabolites, second messengers, neurotransmitters, within living tissues. Another notable feature of optical sensors and actuators is that they can be used to probe physiological processes over a wide range of spatial scales. This article selection particularly draws the attention to this point, demonstrating the application of optical probes from the submicrometric scale of insulin granule mobilization (Hatakeyama et al.), through membrane receptor activation and cytoplasmic downstream signalling (Demby and Zaccolo), to the tracking of cell-cell interactions, such as the recognition of T cell epitopes (Lee et al.), and the investigation of tissue homeostasis, such as the mechanisms regulating cerebral hemodynamics (Iba et al.).

## Original research articles

The original research article by Hatakeyama et al. showed a novel approach to visualize the movement of insulin granules in pancreatic β cells using single molecule analysis with quantum dot-labelled insulin granule membrane proteins. The usefulness of this approach is evidenced by their findings of strict regulation of insulin granules by cytoskeletons, including microtubules and F-actins. Their approach and findings will shed light on cytoskeleton-assisted regulatory mechanism of insulin secretion, which is involved in the pathogenesis of diabetes.

Iba et al. published an original research article investigating a long-debated issue, whether pericytes and venule smooth muscle cells participate in the regulation of cerebral blood flow. Using an outstanding combination of genetical engineering and optogenetics, they showed that these cells have the ability to affect cerebral blood flow. This work provides important clues to solve the issue and a potent tool for manipulating vascular cells with a clear discrimination of mural cell subcomponents.

## Reviews

G protein-coupled receptors (GPCRs) are a widespread family of cell-surface receptors involved in numerous physiological functions, making them a central focus of pharmaceutical research. The article by Demby and Zaccolo introduces the significance of GPCRs, their role in drug development, and emerging complexities related to their investigation, such as receptor conformation and compartmentalisation of signals. This background gives a clear understanding of why a diverse set of bioluminescent and fluorescent biosensors is essential for exploring GPCR mechanisms. These have been employed in pair with a range of imaging techniques, from FRET to two-photon imaging, and their future use as high throughput assays may be critical for developing new and better GPCR-targeted therapies.

The review article by Lee et al. outlined advances in T cell signalling research, particularly the use of genetically encoded biosensors and optogenetic tools. These technologies have provided insight into the real-time dynamics of T cell receptor signalling and how spatiotemporal regulation of signalling pathways governs T cell activation. This finding is particularly relevant for improving chimeric antigen receptor (CAR) T cell therapy and provides a new avenue for optimizing cancer therapy.

In summary, this Research Topic highlights the expanding role and diversification of applications of optical biosensors in fundamental studies addressing the molecular pathways associated with critical physiological processes, including immune responses, hormone secretion and cerebral hemodynamics.

## Future directions

Looking ahead, the development of new genetically encoded indicators, synthetic probes, and imaging techniques, promises to expand the range of molecules that can be visualized in living cells. Moreover, the integration of biosensors with fast-developing technologies such as organ-on-a-chip platforms and transgenic animal models will further enhance our understanding of cellular dynamics in complex biological systems.

From unravelling the complexities of cellular signalling to facilitating the development of new therapeutic approaches, fluorescent indicators will continue playing a pivotal role in advancing both basic and translational research.

